# Proteolysis of Extracellular Matrix in Human Disease 2.0

**DOI:** 10.3390/ijms26062700

**Published:** 2025-03-17

**Authors:** Hironobu Yamashita

**Affiliations:** Department of Pathology and Laboratory Medicine, Pennsylvania State University College of Medicine, Hershey, PA 17033, USA; hyamashita@pennstatehealth.psu.edu

Extracellular matrix (ECM) proteins not only migrate during development and function as a scaffold to which various cells attach but also are known to be substrates of various ECM-degrading proteases outside cells. This processing of ECM proteins by proteases is significantly involved in the progression of many diseases, including cancers and tissue development [1]. This Special Issue, an extension of the Special Issue “Proteolysis of Extracellular Matrix in Human Disease”, contains two articles and four reviews that focus on the role of ECM molecules and extracellular proteases in the development of disorders.

In the research article titled “Increased risk of aortic dissection with perlecan deficiency”, Nonaka et al. established an aortic dissection mouse model by knocking out the Perl gene encoding heparan sulfate proteoglycan (perlecan). In Perl KO mice, thinner and partially broken elastic lamina were observed in the aortic wall and mature elastic fibers failed to develop due to a decrease in colocalization with elastin fiber components (elastin and fibrillin-1) Nonaka et al. Thus, this study proposed that perlecan significantly contributes to aortic dissection development.

In the research article titled “MT1-MMP cooperates with TGF-β receptor-mediated signaling to trigger SNAIL and induce epithelial-to-mesenchymal-like transition in U87 glioblastoma cells”, based on the fact that epithelial-to-mesenchymal transition (EMT) is a critical event induced by TGF-β signaling during the progression of high-grade glioblastoma, Djediai et al. used U87 human glioblastoma cells and sphere models to induce EMT marker and SNAIL expressions via TGF-β treatment. Furthermore, the authors utilized small pharmacological molecules, including Concanavalin A (an MT1-MMP inducer), galunisertib (a TGF-β receptor kinase inhibitor), AG490 and tofacitinib (JAK-STAT inhibitors), and epigallocatechin-3-gallate (EGCG), in conjunction with gene silencing and the siRNA targeting of MT1-MMP to assess via this model the role of MT1-MMP in EMT marker expression induced by TGF-β Djediai et al. These results showed the cooperative contribution of MT1-MMP in the TGF-β-induced and EMT-like processes of human glioblastoma.

In addition to these two research articles, three reviews on ECM and proteolysis are published in this Special Issue. In the review titled “The Proteolysis of ECM in Intervertebral Disc Degeneration”, Liang et al. addressed the role of ECM molecules and ECM dysregulation by proteases in lower back pain caused by intervertebral disc degeneration. Moreover, this review described the possibility that the prevention of ECM processing by proteases can be a therapeutic alternative intervention in the cure for intervertebral disc degeneration Liang et al.

Trinh et al.’s review titled “The Role of Matrix Proteins in Cardiac Pathology”, focuses on both ECM molecules and ECM regulatory molecules, as they play a critical role in cardiac development after birth and in homeostasis and tissue repair of the heart. The myocardium consists of various cells, including blood, cardiomyocytes, endothelial cells, and fibroblasts, which secrete various ECM and ECM regulatory molecules. This review addresses the role of ECM proteins (mainly thrombospondin, SPARC, Osteopontin, TenascinC, Periostin, and the CCN family) in cardiovascular events (fibrosis, cardiomyocyte hypertrophy, angiogenesis, and inflammation) and provides information regarding the role of MMPs in ECM remodeling of the myocardium Trinh et al.

Mayfosh et al.’s review article titled “The Heparanase Regulatory Network in Health and Disease”, focuses on heparan sulfate proteoglycans (HSPGs), which are large ECM molecules that contain core proteins and glycosaminoglycan heparan sulfate, interact with other ECM molecules and growth factors, and play a significant role in many biological processes and cancers. Heparan sulfate is regulated by an enzyme named heparinase. Therefore, heparinase expression is critical in many biological processes as well. This comprehensive review provides information on transcription factors, growth factors, cytokines, hormones/metabolites, pathogens, and therapies to regulate heparinase expression, as well as information on how heparinase itself regulates gene and protein expression and protein phosphorylation Mayfosh et al.
